# Decreased resting and nursing in short-finned pilot whales when exposed to louder petrol engine noise of a hybrid whale-watch vessel

**DOI:** 10.1038/s41598-021-00487-0

**Published:** 2021-11-11

**Authors:** P. Arranz, M. Glarou, K. R. Sprogis

**Affiliations:** 1 Department of Animal Biology, Edaphology and Geology, University of La Laguna, BIOECOMAC, Tenerife, Spain; 2 Department of Biology, Aarhus University, Zoophysiology, Aarhus, Denmark; 3 Húsavík Research Centre, University of Iceland, Húsavík, Iceland

**Keywords:** Behavioural ecology, Conservation biology, Animal behaviour

## Abstract

Vessel noise is a primary driver of behavioural disturbance in cetaceans, which are targeted during whale-watch activities. Despite the growing, global effort for implementing best-practice principles, to date, there are no regulations on whale-watch vessel noise levels. Here, we test the hypothesis that a whale-watch vessel with a low noise emission will not elicit short-term behavioural responses in toothed whales compared to a vessel with a louder engine. We measured behavioural responses (n = 36) of short-finned pilot whales (*Globicephala macrorhynchus*) to whale-watch vessel approaches (range 60 m, speed 1.5 kn). Treatment approaches with a quieter electric engine (136–140 dB) compared to the same vessel with a louder petrol engine (151–139 dB) (low-frequency–mid-frequency weighted source levels, re 1 µPa RMS @ 1 m) were examined. Focal whales were resting mother and calves in small group sizes. During petrol engine treatments, the mother’s mean resting time decreased by 29% compared to the control (GLM, p = 0.009). The mean proportion of time nursing for the calf was significantly influenced by petrol engine vessel passes, with a 81% decrease compared to the control (GLM, p = 0.01). There were no significant effects on behaviour from the quieter electric engine. Thus, to minimise disturbance on the activity budget of pilot whales, whale-watch vessels would ideally have source levels as low as possible, below 150 dB re 1 µPa RMS @ 1 m and perceived above ambient noise.

## Introduction

Commercial whale-watching is a growing, multi-billion-dollar tourism industry globally^1,2^. In 2009, 13 million people participated in whale-watching tours across 119 countries and overseas territories, spending more than USD $2.1 billion^3^. The growth of whale-watching globally brings a subsequent rise in the number and/or size of vessels used to watch cetaceans (whales, dolphins, porpoises). Whale-watching has been viewed as a non-invasive activity, and an economic alternative to whaling^4,5^. Whale-watch vessels, however, spend large amounts of time within close proximities of cetaceans, and consequently, can have adverse fitness consequences on targeted individuals or populations^6^. An increase in the presence of whale-watch vessels was related to a decrease in the relative abundance of bottlenose dolphins (*Tursiops aduncus*) in Shark Bay, Australia^7^, while in Doubtful Sound, New Zealand, there was an overall decrease in population size of bottlenose dolphins (*T. truncatus*)^8^. Exposure to vessel noise can elicit behavioural responses in killer whales and cause a temporary hearing threshold shift^9^. Furthermore, Sprogis et al*.*^10^ determined that the short-term behavioural response of resting humpback whales (*Megaptera novaeangliae*) is driven by underwater vessel noise level. For example, humpback whales on their resting ground off Exmouth, Australia, reduce the proportion of time spent resting in the vicinity of a noisy vessel by 27% compared to a quiet vessel^10^. Underwater noise is an important metric to consider, as hearing is the primary sensory modality of cetaceans. Sound travels fast underwater, and thus, cetaceans use sound for communication, sensing predators and locating food^11,12^. Although cetaceans are affected by underwater vessel noise, through behavioural changes^10^ and call repertoire changes^13–15^, the noise levels from whale-watch vessels are not considered in existing guidelines.

Anthropogenic underwater noise is increasing globally^16^ and the primary source is vessel noise^17,18^. Baleen whales have been considered to be mostly affected by the lower frequencies of vessel noise^19–21^. However, mid-frequency and high-frequency specialists are also affected by vessel noise^22,23^. Elevated ambient noise levels at high frequencies have the potential of masking echolocation and communication signals of toothed whales, such as porpoises and beaked whales^15,23,24^. Thus, vessel noise during whale-watching should be considered for both baleen whales and toothed whales.

The source levels (SLs) of whale-watch vessels moving at slow speed (< 10 kn; typically during whale-watching) range from 138 to 169 dB re 1 μPa @ 1 m^25,26^. How cetaceans respond to whale-watch vessel noise depends on the vessel sound characteristics, along with the style of driving, angle of approach and duration of time approaching targeted species. Thus, to minimise the impact on cetaceans, regulatory bodies have guidelines on the distance, speed and angle of approach of the vessel and number of vessels permitted to watch the same group of cetaceans^26^. However, despite the increasing push by regulators for best-practice principles or codes of conduct, to date, there are no regulations on vessel noise levels. Since current whale-watching guidelines assume that physical proximity is the primary driver of disturbance of cetaceans^27^, a quiet vessel is assumed to have the same impact with that of a very noisy vessel which is at the same distance, angle of approach and speed. In reality, quieter vessels have been proven to minimise behavioural reactions of baleen whales compared to noisier vessels^10^.

To facilitate the sustainability of the whale-watching industry and to support the development of best practice guidelines based on noise level, we tested the hypothesis that a whale-watch vessel with a low noise emission will not elicit short-term behavioural responses in toothed whales compared to a vessel with louder noise emissions. To do this, we measured behavioural responses of 36 mother-calf short-finned pilot whales (*Globicephala macrorhynchus*; here in pilot whales) to whale-watch vessel approaches during controlled exposure experiments. During experiments with a hybrid whale-watch vessel, the quieter electric engines were used and behavioural responses of the pilot whales were compared to when the same vessel approached with the louder petrol engines in use. Experiments were conducted in a deep-water environment off the Canary Islands, where ambient noise levels are low, and the noise emission levels of whale-watch vessels are audible to the whales.

## Materials and methods

### Study area and species

**Figure 1:**
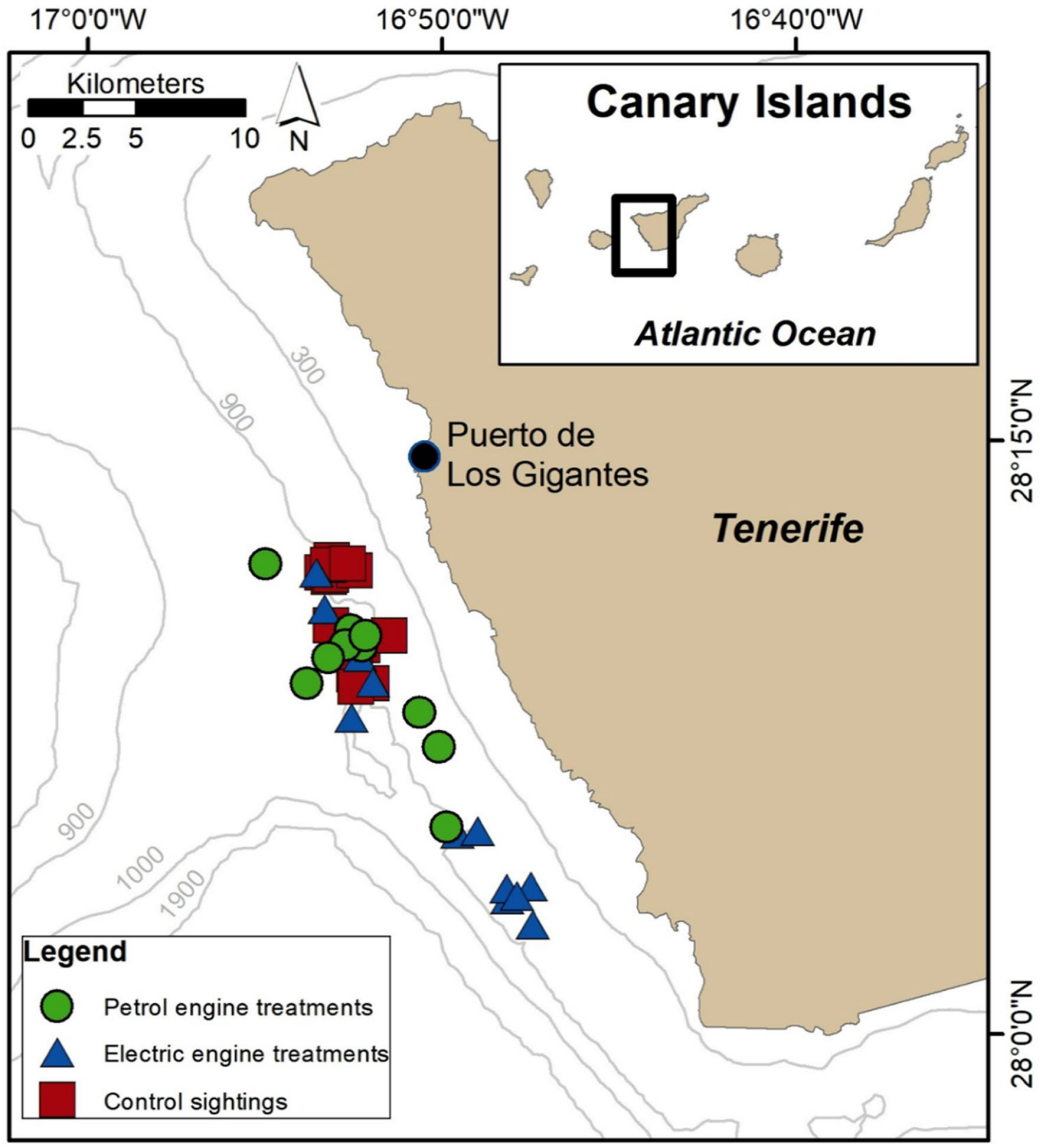
Study area off Tenerife, Canary Islands. Symbols represent control, electric engine and petrol engine treatments. The vessel departed from the Puerto de Los Gigantes marina. Grey lines represent the depth contours of these deep offshore waters (in metres).

Fieldwork was conducted in 2020 and 2021 off the western side of Tenerife, Canary Islands (Spain; Fig. 1), within the special conservation area Franja Teno-Rasca (European Union Natura 2000 Network ES7020017; 28.193200° N, 16.891800° W). The pilot whales off Tenerife are an island-associated population, comprising around 250 resident individuals^28–30^. The area holds generally calm waters on the leeward side of the island, with deep oceanic waters relatively close to shore (Fig. 1). There has been an intense tourism industry since the early 90s, including whale-watching and other recreational activities^31^. The whale-watching tours are focused on the pilot whales as they provide a reliable source of revenue year-round; in 2018, there were 48 operators and 68 vessels operating off Tenerife^32^. Whale-watching has increased so much that the Canary Islands that it is the fourth most common destination for whale-watching globally^33^.

### Noise exposure experiments

Vessel approaches replicating a whale-watch scenario were conducted on resting pilot whale mother-calf pairs. Resting was defined as a low activity level and included whales logging on the surface, near-stationary a few meters below the surface, swimming and/or surfacing slowly (speed < 2 knots). The resting behavioural state offered a standardised behaviour that facilitated detection of noise-induced disturbance. Resting of the mother calf pair was confirmed by observation with the aid of 7 × 50 Fujinon binoculars for ~ 5 min. Lactating mothers were defined as an adult whale > 3 m in length, and a calf was defined as a whale < 2/3 length of the adult it was accompanying, being in close contact with and nursing from. Pilot whale calves usually nurse from their mother up to 3 years of age^34^. Experiments were conducted on mother-calf pairs that were in small group sizes, as small groups reduced the potential for behavioural changes being induced from conspecifics in this social toothed whale species. A group of pilot whales was defined as individuals < 100 m apart participating in the same behaviour. Furthermore, mother-calf pairs were the focus of the vessel approaches as lactating mothers are likely the most sensitive to anthropogenic disturbance as they entail the highest metabolic cost through nursing and lactation^35,36^.

Pilot whales exhibit alloparental care (i.e., non-parent whales help to take care of young that are not their own)^37^. Hence, during foraging excursions of the mothers, calves can remain at the surface accompanied by another adult female or juvenile, as observed in long-finned pilot whales (*Globicephala melas*)^38^. Therefore, sampled mother-calf pairs could potentially include non-parent whales. The mother or the non-parent whale were tracked at all times, unless it was not possible (i.e., the adult dove to depth), in which case only the calf was tracked.

**Figure 2:**
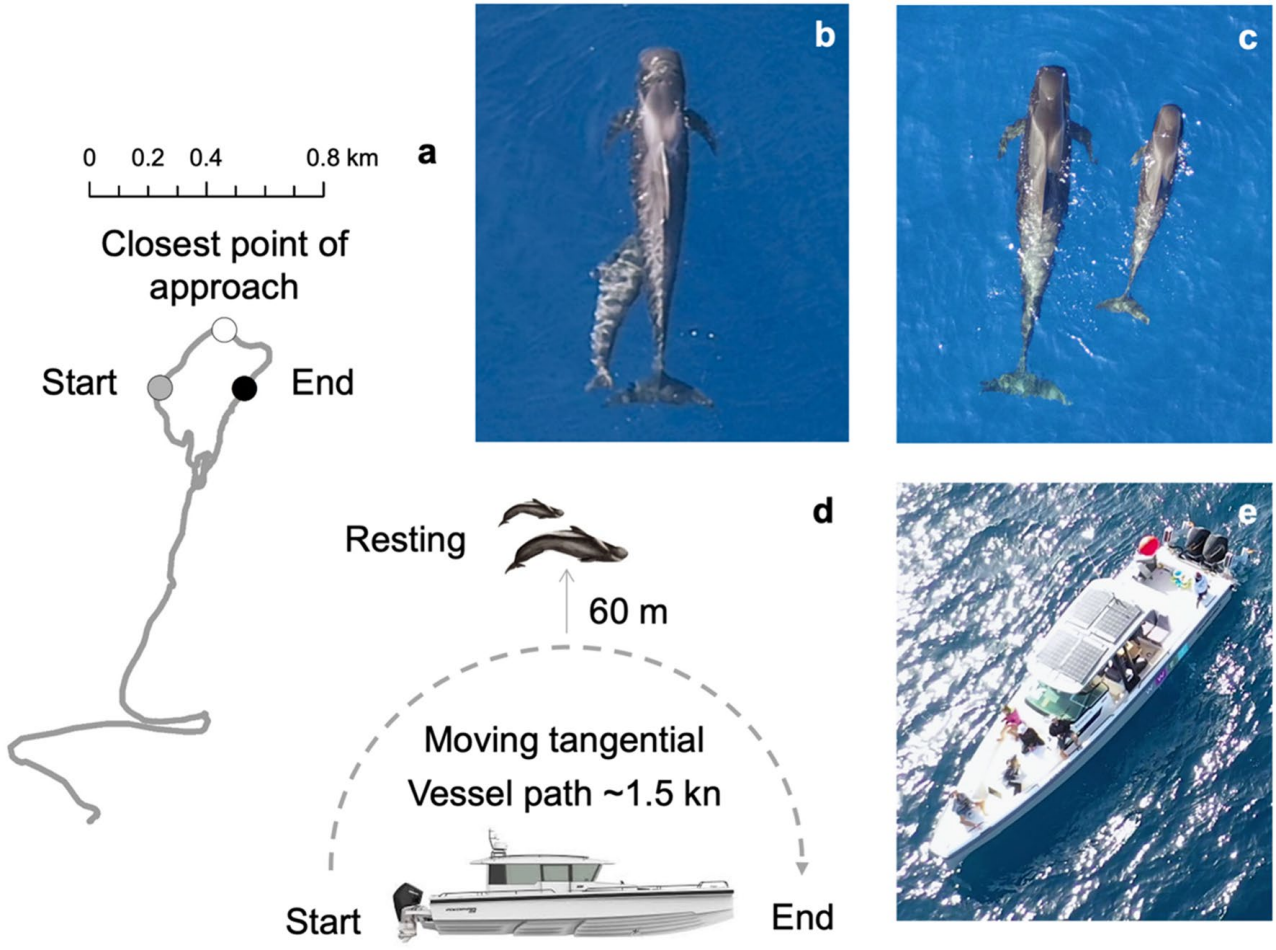
Methodological approach used during the experiments. (**a**) Vessel path recorded during a treatment scenario (example focal follow with electric engine), (**b**,**c**) aerial vantage point of a nursing and resting pilot whale mother-calf pair, (**d**) schematic diagram of treatment (electric and petrol engine) scenarios with 15 min duration approaches beginning > 200 m distance away and transiting in parallel/tangential to the whales; note that during the control scenario the vessel was stationary > 200 m with the engine in neutral, and (**e**) the hybrid whale-watch vessel with electric and petrol engines. Photographs were taken by Patricia Arranz.

**Figure 3:**
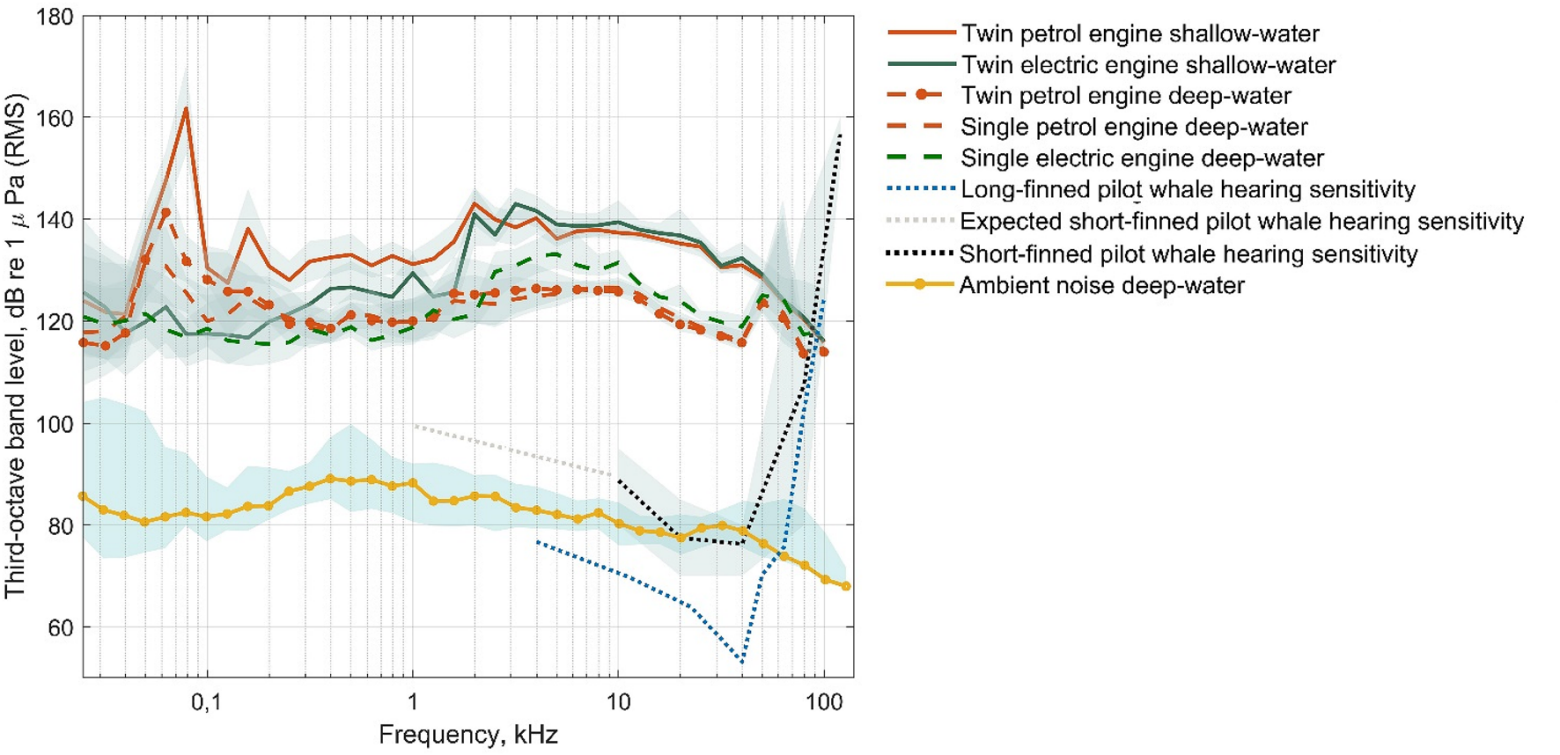
Whale-watch vessel third-octave band levels (TOLs) in dB re 1 µPa (RMS) for single and twin petrol and electric engines in shallow and deep-water habitats off Tenerife. Underwater ambient noise TOLs in a deep-water habitat off Tenerife (~ 1000 m water depth) were measured from SoundTraps at 4 and 400 m depth. The shaded regions represent the 95% confidence intervals. The audiogram of the short-finned pilot whale (*Globicephala macrorhynchus*) for tested frequencies ranging 10–120 kHz^41^ is represented as a dotted black line. The expected hearing sensitivity of the short-finned pilot whale at 1–10 kHz proposed by the authors is shown as a grey dotted line. The expected hearing sensitivity is based on the low frequency vocalisations described for this species^43^ and the described hearing sensitivity of a species within the same genus, the long-finned pilot whale (*Globicephala melas*)^44^.

The experiments were conducted from a small commercial whale-watch vessel (Axopar 37-foot rigid hull, www.whalewise-ecotours.com/about/boat) powered by either two Torqeedo 11 hp electric engines or two 4-stroke Mercury 250 hp outboard petrol engines (Fig. 2). A *control* and *treatment* experimental design was applied, where the behaviour of the whales were recorded for around 15 min each under three possible scenarios: (1) *control group*, where there was an absence of the vessel (> 300 m stationary in neutral), (2) *treatment group with electric engine*, where the vessel transited past the pilot whales in a whale-watch approach (Fig. 2a), and (3) *treatment group with petrol engine*, where the vessel transited past the pilot whales in a whale-watch approach (Fig. 2a). Control and electric engine treatments were conducted in November 2020, whereas petrol engine treatments were conducted in March 2021 due to time constraints. The treatment vessel approaches began when the vessel started moving in gear, and this was considered as time 0. Whale-watch vessel approaches resembled the whale-watching guidelines for the Canary Islands, where a skipper can approach pilot whales from the side to 60 m distance < 4 kn speed (Canary Islands Government 2000, Spanish Government 2007). During the electric and petrol engine treatments, the skipper approached tangentially from the rear starting at 200 m away, and passed by the mother-calf pair at a slow speed (1.5 kn) and 60 m distance (> 40 m and < 70 m range, max ~ 30 dB difference), ending at 200 m away from the group (Fig. 2d). The distance from the vessel to the mother-calf focal pair was estimated using a Busnell Pro Rangefinder (Bushnell, Kansas City, MI, USA). During all experiments, the echosounder was switched off to limit external explanatory variables as it may affect the heading or behaviour of the pilot whales^39^.

A minimum of 10 replicates were aimed to be achieved for each scenario. With this experimental setup, the effect of ‘individual’ was not accounted for between the control and treatment groups but the large sample size (> 10 mother calf pairs) diluted the ‘individual’ effect (e.g., individual whales will respond differently). To ensure that the same mother-calf pair was not sampled twice, and that other nearby pilot whale groups were not exposed to the same noise, replicates on the same day were taken at least ~ 1 km away from the previous experiment. Moreover, sampled pilot whales were individually identified using pictures of their dorsal fin taken with a digital SLR camera (Canon 60D equipped with a 300 mm zoom lens)^40^. A photo-identification catalogue was created and the shape of the dorsal fin of every sampled individual was compared visually.

### Vessel and ambient noise levels

Reference^26^ measured the underwater noise level of the whale-watch vessel powered by both the twin electric and petrol engines when transiting at low speed (< 4 kn) in shallow inshore waters (i.e., < 25 m depth) within the same study area, using a SoundTrap (288 kHz sampling rate, 16 bit, flat (± 2 dB) frequency response from 0.02 to 120 kHz, clip level 175 dB re 1 μPa), suspended at 4 m from a weighted rope. Using the same methodology, additional vessel noise recordings were made in 2020 in deeper offshore waters, close to pilot whale sighting locations (28.249833° N, 16.864766° W) to mimic the habitat conditions where pilot whales are exposed to vessel noise. In this case, the source level of one electric engine was measured as there was a failure of the other electric engine after conducting the vessel approaches (with the two electric engines together), which prevented simultaneous recording of noise levels of both electric engines. Single electric engine and twin petrol engine noise measurements were taken for SL comparison.

Pilot whales are mid-frequency (MF) specialists with some sensitivity towards the lower frequencies, with best hearing in the range from 10 to 50 kHz^41^. Vessel noise was frequency-weighted to match low-frequency (LF) and mid-frequency (MF) weighting^42^. ^26^reported LF and MF weighted source level (SL) of the whale-watch vessel being 136–140 dB and 151–139 dB re 1 μPa, for twin electric and petrol engines in shallow waters, respectively. Ambient noise third-octave-band levels (TOLs) (2 s time averaging window, Hann window with 50% overlap) were also recorded in the same deep-water area^26^. Ambient noise levels were recorded at ~ 1000 m water depth at (a) ~ 4 m depth from the surface for 5 min periods on the 23th March (28.10534° N, 16.80551° W) and 24th March 2019 (28.16395° N, 16.87595° W), and (b) ~ 400 m depth from the surface for a 5 h period on the 24th March 2019 (28.18306° N, 16.8626° W) as in^26^. Ambient noise levels off Tenerife remain similar across the year^14^, thus recordings from Arranz et al*.*^26^ were suitable to use for this study. Figure 3 represents the whale-watch vessel SL TOLs (shallow inshore and deep-water offshore measurements for single and twin engines and the ambient noise received level (NL) in dB re 1 µPa. Noise levels experienced by the animals during control scenarios were assumed to be equal to the ambient noise statistic.

### Unmanned aerial vehicle focal follow procedure

Behavioural focal follows were conducted on pilot whale mother-calf pairs using unmanned aerial vehicles (UAVs) to record the occurrence of behavioural events. Two quadcopters were used, a DJI Inspire 1 Pro UAV and a DJI Mavic 2 Pro UAV which had a longer flight time (diameter without propellers and weight 560 mm, 3400 g and 354 mm, 907 g, respectively, www.dji.com). The Inspire 1 Pro had a 16-megapixel Zenmuse X5 camera and the Mavic 2 Pro had a 20-megapixel Hasselblad camera (both recorded 4 K video, 3840 × 2160, 30 fps). The UAVs were launched and retrieved by hand from the stern of the whale-watch vessel. The distance between the UAV and the boat was always < 400 m to provide a clear line of sight to the UAV. Calibration of the gyro sensors of the UAVs were conducted on land before flying.

Focal follow of mother-calf pairs during control experiments were performed in two consecutive flights with the Inspire, due to its short flight time, and in a single flight with the Mavic during treatment experiments. The UAV pilot used a live-feed iPad 6th gen (9.7″) tablet, equipped with an anti-glare glass and shade hood, which was connected to the UAV remote controller to locate the whales (following Sprogis et al.^10^). Once the group was located, the UAV was positioned above the focal mother-calf pair with the camera in zenithal position (vertically down) and the recording was initiated via the remote controller. At this time, the vessel started moving in treatment scenarios (~ 15 min duration each) while the UAV hovered above the mother-calf pair at an altitude between 30 and 60 m, to minimise potential noise disturbance by the UAV on the animals^45–47^. UAVs were flown in good weather conditions (wind speed < 10 kn and no precipitation). In addition to video, UAVs logged UTC time, GPS altitude and positioning (WGS84 ellipsoid) every 100 ms.

All experiments were performed in accordance with relevant guidelines and regulations. Data were gathered with ethics authorization of the University of La Laguna Animal Use Ethics Committee. The UAVs were operated under an UAV Operator licence (Register # 2020064914) and an Advanced certificate of aircraft piloted by remote control (RPA20605OT and RPA20605OP) under the Spanish Aviation Safety and Security Agency (AESA). All research was conducted under a research permit from the Spanish Ministry for the Ecological Transition and the Demographic Challenge (permit AUTSPP/41/2020).

#### Data processing of UAV videos

Pilot whale behavioural events were registered from the UAV videos using Solomon Coder (v. beta 19.08.02; https://solomon.andraspeter.com/), following^48^. Behavioural events recorded in the UAV videos were identified from a behavioural ethogram (Table 1). The presence of instantaneous events during a focal follow (presence 1/absence 0) were used in the analyses (Table 1). Additional metrics were selected for further analyses, including, the proportion of time resting, nursing, and diving, and respiration rate. Resting was defined as a behavioural state that included continuous behavioural events with low activity levels; logging on the surface, stationary underwater or moving slowly (< 2 kn with slow surfacings). The proportion of time resting consisted of the sum of resting behavioural events divided by the duration of the focal follow (continuous value between 0 and 1). Nursing was defined as when an infant touched with its rostrum to the genital area of an adult female pilot whale (out of view of the UAV) (Fig. 2b). The infant could be lying near-motionless whilst its mother was resting, or could be slowly swimming if the mother was swimming. Nursing was a continuous behaviour for which the proportion of time nursing was calculated (sum of nursing divided by the duration of the follow, continuous value between 0 and 1). Diving was defined as when the focal whales swam vertically to depths and the edges of the body were difficult to discern for continuous periods (Table 1; diving did not include ‘remaining stationary underwater’). The number of breaths taken by focal mother-calf pairs were registered and the respiration rate was calculated as the number of breaths taken during each focal follow divided by the duration of the focal follow. The number of calf breaths were difficult to detect at an altitude > 40 m; thus, videos were viewed twice to register any missed breaths. A breath was defined as an opening of the blowhole, even if vapour was not visualised, which accounted for shallow exhalations. The duration of focal follows was from the start to end time of UAV video recording, and this time was adjusted individually for mother and calves to subtract the amount of time a focal individual was off frame. If there was more than one mother-calf pair appearing in the frame, the relative mother-calf length ratio was used to distinguish the focal mother-calf pair between consecutive control video recordings. During post-processing, the relative length ratios of all mother-calf pairs present in the group were calculated by extracting a still frame from the UAV footage at the same altitude in which both animals were lying flat at the surface. The length from the tip of the rostrum to the notch in the tail fluke was then measured (in pixels) for each animal and the relative length ratio between each mother-calf pair was calculated and compared for among video recordings to determine the focal pair.

**Table 1:** Behavioural events of pilot whales for Tenerife, based on^49^.

Behavioural event	Definition
Apparent nursing	An infant touched its rostrum to the mammary slit area of an adult female pilot whale (out of view of the UAV). The infant may be lying near-motionless whilst its mother was resting, or may be slowly swimming if the mother was swimming. The infant was parallel and almost under the mother (with both tails facing backwards)
Belly aside	Pilot whale swam on its left or right side, with one pectoral fin vertically directed towards the water surface. In some cases, half of the body was exposed out of the water
Belly to belly	Two pilot whales swam belly-to-belly without touching each other
Belly up	Rolled so that its ventral side was facing the surface of the water. Often the belly was fully exposed out of the water
Body contact	Physical contact between two or more pilot whales by several means, e.g., pectoral fin touches or rubbing body parts
Bubble display	Emitted bubbles from the blowhole underwater. These can be a single bubble, a whole cloud or bubble trains
Diving	Swam straight down vertically to a depth when the edges of the body may be difficult to discern. They may even disappear from the image
Encircling	One pilot whale swum circles around another in a small radius and at relatively high speed
Horizontal roll	A complete roll (360°) along the longitudinal axis and parallel to the water surface
Logging	Remained at the surface motionless (> 5 s)
Milling	Moved slowly at the surface without a fixed bearing
Mouth to mouth	Two or more pilot whales positioned their rostrums towards each other. Sometimes the rostrums were touching
Moving slowly	Swam slowly at the surface or underwater (< 2 knots)
Resting underwater	Remained underwater close to surface near-motionless
Rough housing	An adult pilot whale striked the side of the calf with its head or body
Spyhop	Vertically lifted its head out of the water so that the eyes were completely in the air, with a vertical re-entry
Tail slap	A slap with the ventral side of the tail or tailstock on the water surface. This behaviour can be repetitive with short intervals between slaps
Vertical roll	A complete roll (360°) along the ventral axis and perpendicular to the water surface

#### Data analyses for the behavioural effects on mother-calf pilot whales

The response variables of interest were (1) the proportion of time resting for the mother and calf, (2) the proportion of time nursing for the calf, (3) the presence of instantaneous behavioural events for mother and calf, and (4) the respiration rate of mother and calf (Table 2). Pilot whales rest to conserve energy, and calves nurse to gain energy and grow^50,51^, thus a decrease in resting and/or nursing due to anthropogenic disturbance will alter the natural behavioural budget of the pilot whales. Respiration rate relates to energy expenditure and if there is an increase in the number of breaths taken during a disturbance then this is energy that would not have been expended naturally^10,52^. Energy is needed to invest in nursing young, foraging for prey, socialising with conspecifics, and evading predators^50,53^. Explanatory variables of interest were the whale-watching scenario (control, electric engine, or petrol engine vessel passes), group size of whale pilot whales, and the presence or absence (pres/abs) of behavioural events.

**Table 2:** Models used in analyses to test for behavioural effects of underwater electric and petrol engine vessel noise on pilot whales. Models used were linear models (LM) and generalised liner models (GLM). ^a^Fitted for both mother and calf. The scenario was control, electric engine, and petrol engine vessel passes. Presence and absence (pres/abs) of behavioural events outlined in Table 1.

Response variable	Explanatory variables explored	Type	Error distribution	Link function
Proportion of time resting^a^	Scenario, group size	GLM	Quasibinomial	Logit
Proportion of time nursing for calf	Scenario, group size	GLM	Quasibinomial	Logit
Proportion of time diving for mother	Scenario, group size	GLM	Quasibinomial	Logit
Pres/abs behavioural events	Scenario, group size	GLM	Binomial	Logit
Respiration rate^a^	Scenario, group size, pres/abs behavioural events	LM	Gaussian	Identity

Prior to modelling, data were examined for outliers and individual whales were assumed to have independence/the same probability of reacting to a treatment^54^. There was no collinearity or relationship between scenario, group size and pres/abs behavioural events. Linear models were constructed in *R* v1.1.463^55^. To conform to model assumptions of normality, the response variable of maternal respiration rate was log-transformed (log_10_). To ensure the fitted values for proportion data (resting, nursing and diving) ranged from 0 to 1 a binomial distribution was used, and to explain more variance in the data a quasibinomial generalised linear model (GLM) was used. Akaike information criterion (AIC) model selection was used to select the most parsimonious models. The presence of instantaneous events for calves was overdispersed and an explanatory variable of interest known to influence behaviours was not available for use (i.e., calf length), thus models were not run.

#### Model validation

Model validation was conducted to test if the regression model assumptions were met. Homogeneity was explored through equal variances in scatterplots with residuals against fitted values, and against treatment and group size explanatory variables. Normality was examined to see if residuals deviated greatly in the Q–Q plot. Influential points were observed through Cook’s Distance to check if there were extreme values above 1. Temporal autocorrelation and partial autocorrelation were examined with correlograms (auto-correlation function plots) to examine if there were values close to 1 dependent on each other. Overdispersion (variance is larger than the mean) was checked by dividing Pearson’s residual deviance with the degrees of freedom, with a dispersion ratio > 1 indicating overdispersion. The final models met these assumptions.

## Results

### Survey effort

Control and electric engine data were collected from 2nd to 8th November 2020, with 66 h on the water. Petrol engine data were collected from 13th to 15th February 2021, with 21 h on the water. Data were collected in daylight hours from 7:30 to 17:00 h local time. 19 control, 18 electric, and 16 petrol focal follows were attempted. Focal follows were conducted in an average water temperature of 22.2 °C (range 21.1–23.4 °C) in November and 19.3 °C (range 18.0–20.7 °C) in February, and water depth of 854 m (SD = 76). Focal follows for the control data (n = 13; 2.6 h total UAV flight time) had a mean duration of 12.5 min (SD = 0.002). The time intervals between each consecutive control flight, due to changing the UAV batteries, averaged 1.7 ± 0.6 min between landing and take-off and 3 ± 1 min between end and start of video recordings. Selected focal follows for the treatment data with the electric engine (n = 13; 2.5 h total UAV flight time) had a mean duration of 11.6 min (SD = 0.002) and the average closest point of approach was 64 m (SD = 15.6). Selected focal follows for the treatment data with the petrol engine (n = 10; 1.9 h total UAV flight time) had a mean duration of 11.5 min (SD = 0.001) and the average closest point of approach was 55 m (SD = 14.5). Mean group size was 5 whales (SD = 2.2) for control data, 6 whales (SD = 2.9) for electric engine treatments and 7 whales (SD = 3.9) for petrol engine treatments. No repeated mother-calves were photo-identified. For analyses, focal follow durations were adjusted if the mother and calf were not visible in the frame. For the mother, the average video duration was 10.5 min (SD = 4.1) for control, 11.2 min (SD = 3.3) for electric engine, and 11.4 min (SD = 2.7) for petrol engine data. For the calf, the average video duration was 11.2 min (SD = 3.3) for control, 11.4 min (SD = 2.7) for electric engine, and 11.1 min (SD = 1.7) for petrol engine data (Table 3).

**Table 3:** Summary of controlled exposure experimental focal follows with an unmanned aerial vehicle (UAV) with mean flight times and standard deviations (in brackets). The closest point of approach (CPA) of the whale-watch vessel is listed, and the minimum and maximum range to the animals, along with the mean group size of pilot whales.

Type	n	Total UAV flight time (h)	Mean flight duration (min)	Average CPA (m)	Range (m)	Mean group size (n)
Control	13	2.6	12.5 (0.002)	NA	200	5 (2.2)
Electric	13	2.5	11.6 (0.002)	64 (15.6)	54–115	6 (3.0)
Petrol	10	1.9	11.5 (0.001)	55 (14.5)	35–71	7 (3.9)

#### Proportion of time resting for mother-calf pairs

**Figure 4:**
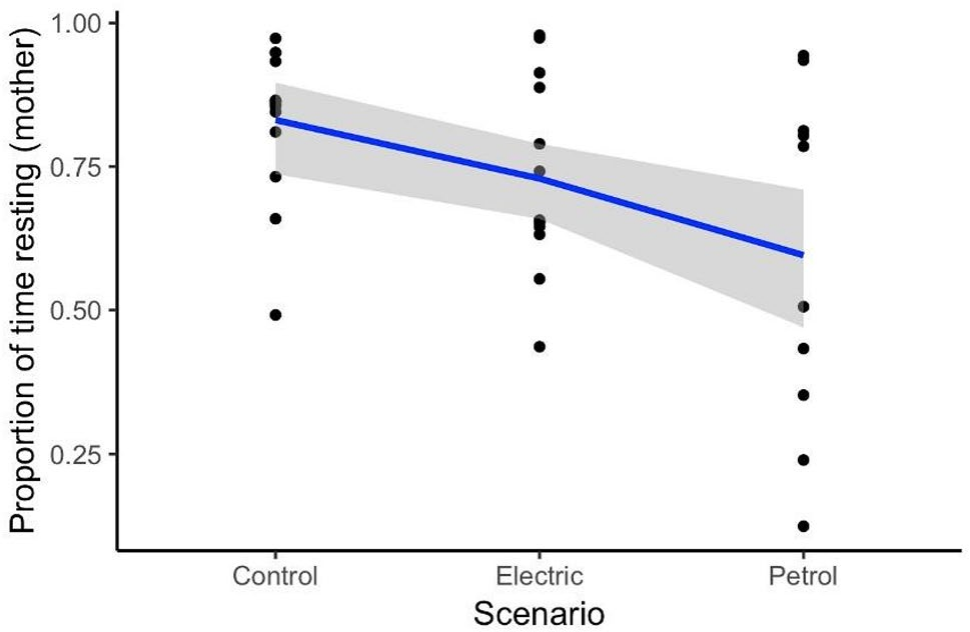
Proportion of time resting for pilot whale lactating mothers as a function of different whale-watching scenarios: control (without a vessel pass), electric engine passes and petrol engine passes (black dots). The solid line represents the fitted values of the linear model, and the shaded area represents the 95% confidence interval.

Resting mothers were targeted for focal follows, and during the control data mothers were resting for a large proportion of time (mean = 0.83, SD = 0.13, range = 0.49–0.97). During the treatment with the electric engine, the average proportion of time resting for the mother decreased by 12% compared to control (mean = 0.73, SD = 0.54, range = 0.44–0.98). During petrol engine approaches, the average proportion of time resting for the mother decreased by 29% compared to the control (mean = 0.59, SD = 0.30, range = 0.12–0.94). The proportion of time resting was influenced by the treatment type, with petrol engine passes significantly decreasing the proportion of time resting (GLM: prop.resting.mother ~ scenario, t = − 2.76, p = 0.009; Fig. 4). The proportion of time resting was not significantly influenced by group size (GLM: prop.resting.mother ~ group.size, t = − 1.61, p = 0.12).

Calves rested for a lesser amount of time compared to the mothers. In the control data, the average proportion of time resting was 0.54 (SD = 0.24, range = 0.13–0.89). During the treatment with the electric engine, the average proportion of time resting for the calf was 0.54 (SD = 0.24, range = 0.12–0.85). During the treatment with the petrol engine, the average proportion of time resting for the calf was 0.43 (SD = 0.28, range = 0.12–0.87). The proportion of time resting for the calf was not significantly influenced by the electric engine or petrol engine (GLM: prop.resting.calf ~ scenario, electric t = − 0.08, p = 0.94, petrol t = − 1.06, p = 0.30), or group size (GLM: prop.resting.calf ~ group.size, t = − 0.48, p = 0.63).

#### Proportion of time nursing for the calf

**Figure 5:**
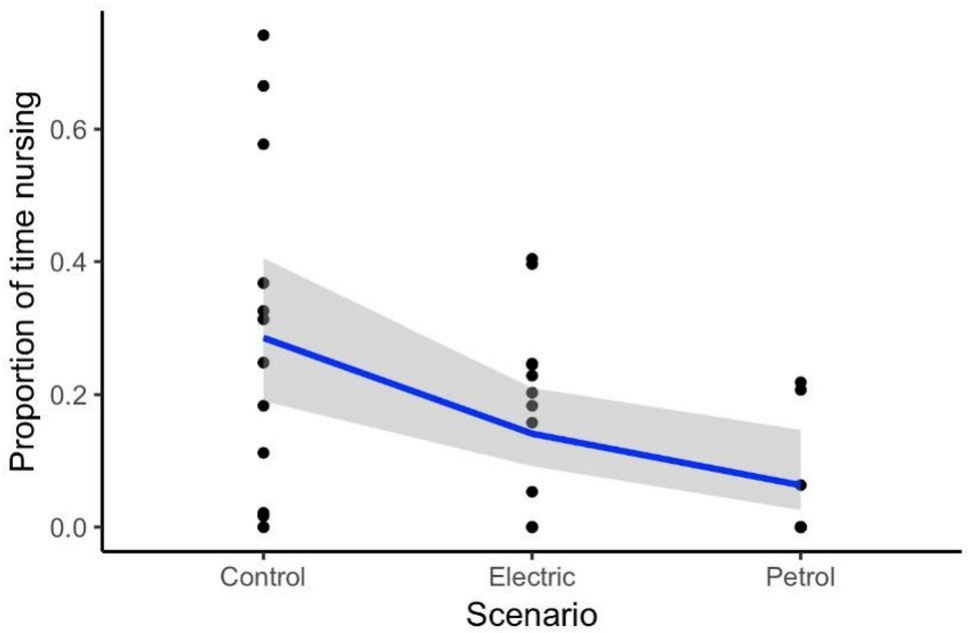
Proportion of time nursing for pilot whale calves as a function of different whale-watching scenarios: control (without a vessel pass), electric engine passes and petrol engine passes (black dots). The solid line represents the fitted values of the linear model, and the shaded area represents the 95% confidence interval.

Nursing was observed from the calf whilst the mother was logging on the surface, stationary underwater, moving slowly, and diving. The average proportion of time nursing for the calf, whilst the mother was predominantly resting, during control data was 0.27 (SD = 0.26, range = 0–0.74). During passes with the electric engine, the average proportion of time nursing was 0.16 (SD = 0.15, range = 0–0.40). During passes with the petrol engine, the average proportion of time nursing decreased by 81% compared to the control (mean = 0.05, SD = 0.09, range = 0–0.22). The proportion of time nursing was significantly influenced by petrol engine vessel passes, with petrol engine passes significantly decreasing the proportion of time nursing (GLM: prop.nursing.calf ~ scenario, t = − 2.70, p = 0.01; Fig. 5). The proportion of time nursing was not significantly affected by group size (LM: prop.nursing.calf ~ group.size, t = 0.03, p = 0.98).

#### Proportion of time diving for the mother

During the control data, mothers were diving for an average proportion of time of 0.10 (SD = 0.14, range = 0–0.50). During the treatment with the electric engine, the average proportion of time diving for the mother was 0.20 (SD = 0.15, range = 0–0.53). During petrol engine approaches, the average proportion of time diving for the mother increased nearly three-fold compared to the control (mean = 0.29, SD = 0.25, range = 0–0.74). The proportion of time diving was not significantly influenced by scenario (t = 1.99, p = 0.06), however it was influenced by an increase in group size (GLM: prop.diving.mother ~ scenario + group.size, t = 2.05, p = 0.05).

#### Occurrence of behavioural events for mother-calf pairs

The occurrence of behavioural events (Table 1) across focal follows for the mother were limited (n = 16 total, present in 11 of 36 focal follows, 30.6%). The mother performed events including spyhop, vertical roll, rough housing, belly aside, belly up and body contact. The most common behavioural event was body contact (n = 6 times across 5 mothers). The least common behaviour that was only performed once was vertical roll. Tail slap, horizontal roll, mouth to mouth, belly to belly, bubble display and encircling were not observed. Behavioural events were present in the control (n = 10) and electric engine (n = 6) scenarios but not the petrol engine scenario. The occurrence of behavioural events were not significantly influenced by scenario (GLM: pres.abs.beh.mother ~ scenario, electric t = − 1.20, p = 0.24, petrol t = − 0.01, p = 0.99), or group size (GLM: pres.abs.beh.mother ~ group.size, t = − 0.16, p = 0.87).

The calf performed several behavioural events (n = 137 total, present in 24 of 36 focal follows, 66.7%), including bubble display, horizontal roll, mouth to mouth, belly aside, spy hop, belly aside, belly up and body contact. The most common behavioural event was body contact with the mother predominantly (n = 43 times across 15 calves). The least common behaviour was vertical roll, and mouth to mouth with another conspecific (n = twice each). Belly to belly and encircling were not observed. Behavioural events were present in the control (n = 38), electric engine scenario (n = 56) and the petrol engine scenario (n = 43).

#### Respiration rate for mother-calf pairs

The respiration rate for mothers in the control data was 2.74 breaths min^−1^ on average (SD = 1.14, range = 1.68–5.25). During the treatment with the electric engine, the mother’s respiration rate was 2.53 breaths min^−1^ on average (SD = 0.99, range = 1.56–4.88). During the treatment with the petrol engine, the mother’s respiration rate was 2.50 breaths min^−1^ on average (SD = 1.40, range = 1.28–5.31). Treatment scenarios did not significantly affect maternal respiration rate (LM: log(respiration.rate.mother) ~ scenario, F_2,33_ = 0.37, p = 0.70, R^2^ = 0.02). However, this result may have been confounded as the most parsimonious model for maternal respiration rate was partially explained by group size and the occurrence of behavioural events (LM: log(respiration.rate.mother) ~ group.size + behavioural.events, F_2,33_ = 5.24, p = 0.01, R^2^ = 0.24). With an increase in group size, there was a decrease in maternal respiration rate (t = − 2.68, p = 0.012).

Calf respiration rate in the control data was 2.61 breaths min^−1^ on average (SD = 1.10, range = 1.10–4.63). During the treatment with the electric engine, the calf’s respiration rate was 2.69 breaths min^−1^ on average (SD = 0.91, range = 1.56–4.26). During the treatment with the petrol engine, the calf’s respiration rate was 2.45 breaths min^−1^ on average (SD = 0.97, range = 1.44–4.53). Respiration rate was not significantly influenced by scenario (LM: respiration.rate.calf ~ scenario, F_2,33_ = 0.16, p = 0.85, R^2^ = 0.01) or group size (LM: respiration.rate.calf ~ group.size, F_1,34_ = 1.33, p = 0.30, R^2^ = 0.04). The most parsimonious model showed that calf respiration rate was influenced by the presence of behavioural events, with an increase in the number of breaths taken as surface activity increased (LM: respiration.rate.calf ~ pres.abs.behav.events.calf, F_1,34_ = 12.83, p = 0.001, R^2^ = 0.27).

## Discussion

Boat-based whale-watching is currently the greatest economic activity reliant upon cetaceans globally^3^. Whale-watch vessel noise has been identified as a driver for short-term behavioural responses on cetaceans^10^. Although most countries have introduced regulations in order to mitigate the impacts of this industry on cetacean populations, current operational guidelines lack noise level emission criteria during whale-watching activities. Here, we test the hypothesis that a vessel with a low source level operating in a standard whale-watch scenario will not elicit short-term behavioural responses on resting pilot whale mother-calf pairs, whereas a louder vessel will. This hypothesis is based on the premise that the spectral signature of the vessel is similar across frequencies within the hearing range of the targeted pilot whales.

To test this, we conducted experiments with control, electric and petrol engine noise treatments during whale-watch vessel passes at 60 m distance from pilot whales. The response variables of interest were the proportion of time resting/nursing/diving, the occurrence of behavioural events, and respiration rate. During control treatments the whale’s experienced ambient noise levels with a maximum RL of 90 dB re 1 μPa RMS. For the electric engine treatments, both the lower and higher frequency components of the engine noise were at least 50 and 46 dB above ambient noise and likely audible to the pilot whales (LF and MF weighting ranges 0.2–19 kHz and 8–110 kHz, respectively^56^. Accordingly, the electric engine noise had an average LF and MF-weighted SL of 136 dB and 140 dB re 1 μPa RMS. For electric engine passes, there were no significant behavioural responses recorded for mother-calf pairs. In contrast, during petrol engine passes, the mothers’ behaviour was significantly affected by vessel approaches as their resting time decreased by 29% compared to control treatments. Furthermore, the nursing time of the calves decreased by 81% during petrol treatments compared to controls. Pilot whales were likely disturbed within the petrol engine treatments as the lower frequency components of vessel noise were at least 61 dB above ambient noise and were likely audible given the expected hearing range spectrum of the pilot whales (Fig. 3).

Results showed that the electric and petrol treatments did not significantly affect maternal respiration rate. However, this result may have been confounded as the most parsimonious model for maternal respiration rate that was partially explained by group size (i.e., with an increase in group size there was an increase in the number of breaths) and the occurrence of behavioural events. Similarly, calf respiration rate was not significantly affected by treatment scenarios, though the most parsimonious model for calf respiration rate was partially explained by the occurrence of behavioural events. These findings are not surprising, as pilot whales are a social group-living species, and their movements may be affected by conspecifics^57^. Synchronous breathing reinforces social bonds and has been suggested as a response to disturbance in bottlenose dolphins^58^, killer whales (*Orcinus orca*)^59^ and long-finned pilot whales^60^. In the later species, synchrony increases with larger group size and decreases with the occurrence of social events. It is important to note that in baleen whales it has been shown that whale-watching activities have an effect on the respiration rate of whales^10,52^, however for baleen whales it is simpler to tease apart such effects, as focal individuals may be able to be targeted when not in larger groups. As pilot whales are a group-living social species, it is difficult to observe focal individuals solely to examine the effects of vessel approaches without conspecifics. However, during whale-watching tours pilot whales are generally encountered as groups in the wild, so the effects shown here mirror representative whale-watch encounters.

Control and electric engine treatments were conducted in November (autumn), and petrol engine treatments were conducted the following March (winter), however a seasonal effect is not expected to have an impact in the behavioural variables under investigation. As such, pilot whales tagged with digital recording tags off Tenerife (n = 53 whales sampled in October and March) exhibit a stereotyped foraging and dive behaviour without seasonal variability^61^. During the day, this species feeds at 800–1000 m depth in a stable layer of non-migrant, deep-water organisms. At night, the whales dive shallower to feed on mesopelagic organisms that migrate close to the surface at dusk^61,62^. Night-time dives during full moon periods have been reported to be 48% deeper and 17% longer than day-time dives in Hawaiian pilot whales^63^. This could potentially affect the time that whales spend resting at the surface at night, however, our study was conducted during day-time hours and in quarter moon phases, therefore prey-driven variations in resting time between sampling seasons are expected to be small. Pilot whales are non-capital breeders with reproduction occurring throughout the year^57,64^. Therefore, significant seasonal variations in pilot whale calving and nursing rates are not expected in this subtropical habitat, where relatively small sea surface temperature variation occurs between autumn and winter^65^. In this study, there was a limited range in sea surface temperature from 18.0 to 23.4 °C (~ 5 °C maximum difference). In terms of predators, killer whales prey on pilot whales^66,67^. However, these predators are transient visitors in the Canary Islands, arriving early summer following the migration of their main prey, Atlantic bluefin tuna (*Thunnus thynnus*), and leaving the archipelago before autumn^67^. Given that this study was conducted in late autumn and winter, it is therefore unlikely that the presence of natural predators will be the cause of the observed differences in the pilot whale behaviour. Furthermore, no sighting records of killer whales occurred during the duration of the study. Almunia et al*.*^68^ monitored whale-watching effort in SW Tenerife using AIS and results were comparable across 2016–2020 for autumn and winter months. This suggests that differences found in pilot-whale behaviour between control and petrol engine treatments are not motivated due to an increase of whale-watching effort in this area in winter. Further experiments performed with controls in closer temporal association to experimental treatments will strengthen these results.

The electric and petrol engines used in this study had similar spectral signatures and noise levels within the described region of best hearing of the pilot whales, ranging 10 to 50 kHz^41,69^ (Fig. 3). Moreover, the same vessel and approaching procedure were used across treatments. Therefore, one can expect that both the electric and petrol treatments would affect the pilot whales similarly. However, significant responses consistent with behavioural changes were recorded under petrol engine treatments and not electric engine treatments. Spectral signatures of the engines differ in the low frequency range, below 2 kHz (Fig. 3), being on average 10 dB higher for the petrol engine compared to the electric engine. Hearing sensitivity of this species has only been tested above 10 kHz and no information is available on their hearing in the lower frequencies. However, pilot whales produce four main types of communication signals: low- and medium-frequency calls (median fundamental frequency: 1.7 and 2.9 kHz) and two-component calls (median frequency of the low and high frequency components: 2 and 9 kHz)^43^. Therefore, it is expected that the species has relatively fair hearing sensitivity below 10 kHz, likely with a slight slope in the lower frequencies compared to the steep slope of the high frequency region, similarly to long-finned pilot whales^44^. Hence, it is reasonable to consider that pilot whales may be responding to the higher SL of the petrol engine within the low frequency range. Further experiments describing the full audiogram of this species are required to better understand their hearing sensitivity to lower frequency sounds.

### Implications for pilot whale energy budget and fitness

The results presented here are consistent with behavioural alterations leading to an increase of energy consumption by mothers due to reduced resting periods, and to a reduction of energy gained by calves due to reduced nursing. Similarly, the amount of time resting decreased in the presence of whale-watch vessels for Risso’s dolphins (*Grampus griseus*) off the Azores^70^ and bottlenose dolphins (*T. truncatus*) in New Zealand^71^. Since nursing usually occurs during resting periods, disturbance has been proposed to reduce nursing time in cetaceans^6,72^. In pilot whales, energy is required for socialising, foraging, being alert to predators (such as killer whales), and nursing^50,61,73,74^. Such disruptions in the activity budget could lead to long-term effects, such as alterations in the relative abundance of the population, as reported in other cetacean species^7,8^. Pilot whales, like other odontocetes, have developed alloparental care as an evolutionary response to threats of predation on individuals^38,75^. Members of the group termed “babysitters” remain at the surface with younger individuals not yet able to dive to depth while the lactating mothers and other adults go on deep foraging dives. Allonursing (nursing from babysitters) has been documented in toothed whales, including sperm whales^76^ and captive belugas^77^. Therefore, calves may not need to concentrate periods of nursing only when they are in close contact with their mothers. Either way, vessel disturbance during nursing or allonursing periods may affect the female-calf energy transfer and growth of the calves, and ultimately could affect calf survival.

Pilot whales dive to access deep-water prey during the daytime, whereas during the night they exploit prey from the deep scattering layer that migrate at shallower depths^61^. This is part of a behavioural strategy that enables these animals to gain the highest net energy return in the available foraging patches. Resting periods at the surface during the day may be critical for this species to recover from deep-dives and to conserve energy^78^. Alteration of the time resting at the surface after a deep dive may have an impact on the foraging performance of the individuals, either due to oxygen stores not being fully replenished before the next dive and/or as longer periods at the surface may be required for balancing oxygen metabolism^79,80^. This can have long-term consequences on individual fitness and may lead to reduced time available for feeding (either in terms of time at depth or in number of dives per day) and increasing energy consumption due to higher metabolic costs of maintaining higher activity levels.

### Implications for management of whale-watching

Short-term disturbance effects from boat-based whale-watching has been widely reported in cetaceans^6,81^. Thus, the introduction of guidelines or regulations for whale-watching has been the most common method to mitigate the impacts of whale-watching (e.g., including proximity, speed and approach type^3^). However, this study demonstrates that different vessel noise levels can elicit different behavioural responses in toothed whales consistent with behavioural alterations, even if operators comply with current guidelines. The possibility that behavioural responses will affect fitness depends on the ability of the individuals to compensate and varies accordingly to specific ecological and social conditions^81^. Population-specific studies should address the potential long-term consequences of these behavioural responses and inform management of the whale-watching industry^6^. The SL of commercialised whale-watch vessels within a fleet would ideally be recorded with a region. In developed countries, such as the USA, Canada, Australia and Canary Islands, which include the world’s top four whale-watch locations, this could be achievable. It is recommended that vessel SLs be low and perceived above ambient noise. A suggested noise level for vessels operating around baleen whales and toothed whales as close as 100 m is LF-weighted SLs no more than 150 dB re 1 uPa RMS @ 1 m^10^. At closer whale-watching distances, e.g., 60 m in the Canary Islands, the SL of the vessel is recommended to be lower than 150 dB due to the closer proximity imposed. Furthermore, the Canary Islands has quiet ambient noise conditions (Fig. 3^26^, thus in these conditions it is recommended that vessel noise is reduced further to lower the excess noise difference.

The electric engines were quieter compared to petrol engines used in this study and they did not cause behavioural effects on pilot whales. Source levels of the electric engines were below LF < 150 dB, which is a suggested source level for whale-watching guidelines^10^. The same electric engines were the quietest of several whale-watch vessels recorded in a recent study^26^ and remain as a promising low-noise alternative for vessel operations with a potential benefit for the cetaceans^82^. Further studies examining the effects of different types of electric engines on cetaceans are therefore required, to support the implementation of guidelines for green transformation and blue growth pathways for all-electric nautical tourism and shipping. Several vessels have been converted to electric engines globally (e.g., whale-watch vessels in Iceland and the Canary Islands and ferries in Europe), or to quieter engines in general^83,84^. These quieter engines are therefore recommended for use during whale-watch activities, when spending a large amount of time in close proximity and targeting cetaceans (e.g. whale-watching and swim-with-cetacean tours^85,86^).

## Conclusion

The results of this study are consistent with behavioural changes in response to vessel noise leading to an increase of energy consumption by mothers and to a reduction in the energy gain by the calves. The louder petrol engine induced a significant decrease in the proportion of time resting for the mother, and the proportion of time nursing for the calf. Conversely, the quieter electric engine did not cause any measurable behavioural responses compared to the control and petrol engine treatment. These results demonstrate that different vessel noise levels can elicit different behavioural responses on cetaceans, even if operators comply with the current, national whale-watching guidelines. The establishment of SL criteria of whale-watch vessels is recommended, to incorporate into best-practice whale-watching guidelines. Lower whale-watching vessel engine noise will benefit tourists seeking an eco-viewing opportunity, whilst reducing disturbance to cetaceans, ultimately assisting in the sustainability of the multi-million-dollar whale-watching tourism industry.

## Data Availability

The datasets generated during and/or analysed during the current study are available from the corresponding author on reasonable request.
